# Oxytocin reduces neural activation in response to infant faces in nulliparous young women

**DOI:** 10.1093/scan/nsy080

**Published:** 2018-09-07

**Authors:** Peter A Bos, Hannah Spencer, Estrella R Montoya

**Affiliations:** Experimental Psychology, Utrecht University, the Netherlands

**Keywords:** fMRI, parental care motivation, VTA, striatum, reward, caregiving

## Abstract

Infant faces have distinctive features that together are described as baby schema, a configuration that facilitates caregiving motivation and behavior, and increases the perception of cuteness. In the current functional magnetic resonance imaging (fMRI) study, we investigated the effect of a within-subjects intranasal oxytocin administration (24 IU) and caregiving motivation on neural responses to infant faces of varying baby schema in 23 healthy nulliparous women. Overall, infant faces elicited activation in several brain regions involved in reward and salience processing, including the ventral tegmental area (VTA), putamen, amygdala, anterior cingulate cortex (ACC), and insula, and this activation was related to self-reported caregiving motivation. Critically, whereas we hypothesized enhanced neural caregiving-related responses after oxytocin administration, we observed reduced activation in the VTA, putamen and amygdala after oxytocin compared to placebo. In nulliparous women, oxytocin has been shown to reduce neural responses in the same regions in response to social stimuli using other paradigms. Oxytocin might affect neural activation toward social stimuli depending on elicited arousal and personal characteristics. The current study is the first to demonstrate this effect in response to infant faces and thereby adds to specify the role of oxytocin in human social information processing.

## Introduction

As proposed by Lorenz in 1943, more pronounced features characteristic of infant faces, such as a large forehead and eyes combined with a small nose, mouth, and chin, would elicit more caregiving behavior and reduce aggression toward the infant (Lorenz, 1943). Experimental work in the past decades has demonstrated that such a facial configuration, referred to as ‘baby schema’, is related to perceptions of cuteness and positive affective responses (DeBruine *et al.*, 2016), and indeed increases caregiving behavior and motivation, both in parents and in nulliparous adults (Langlois *et al.*, 1995; Glocker *et al.*, 2009a; Hahn and Perrett, 2014). Neuroimaging studies have further shown that baby schema increase activation of several brain regions involved in reward processing, including the ventral tegmental area (VTA), and the ventral striatum (Glocker *et al.*, 2009b; Feldman, 2015; Luo *et al.*, 2015), which might underlie some of these observed behavioral responses. Importantly, the neural reward circuitry is sensitive not only to infant faces but also to endocrine factors including the neuropeptide oxytocin (OXT) that has also been ascribed an important role in the facilitation of parent–infant attachment and caregiving behavior (Rilling and Young, 2014; Bos, 2017; Feldman, 2017). Increasing activation of the neural reward circuitry in response to infant faces is one of the mechanisms by which OXT could facilitate parental caregiving. In the current study, we investigated the effect of OXT on the neural reward circuitry in a group of healthy young nulliparous women when looking at infant faces using functional magnetic resonance imaging (fMRI).

So far, a limited number of studies have directly investigated the role of OXT in neural responses to infant faces, and most of these studies were correlational studies, in which OXT levels were measured from either saliva or blood (Luo *et al.*, 2015; Feldman, 2017). Although there is considerable discussion about the validity of these assessments (McCullough *et al.*, 2013; Leng and Ludwig, 2016), positive relations between OXT levels and attention toward infant faces have repeatedly been found in parents (Luo *et al.*, 2015), as well as enhanced neural reward activation toward infant faces, especially toward own infants *vs* other infants (Strathearn *et al.*, 2009). An important reason to set up the current study, is that only few studies employed OXT administration paradigms. So far, in fathers, increased activation of the caudate (part of the striatum) and the anterior cingulate cortex (ACC) was observed in response to own child pictures after OXT administration (Wittfoth-Schardt *et al.*, 2012; Li *et al.*, 2017), although one of the two studies additionally showed a decreased activation in the more ventral part of the striatum (Wittfoth-Schardt *et al.*, 2012). In women, only one study investigated the effect of OXT administration in response to infant stimuli while using fMRI and showed enhanced activation in the VTA in response to pictures of crying (but not to smiling) infants in both nulliparous and postpartum women (Gregory *et al.*, 2015). Enhanced processing of infant faces after OXT administration was also observed in two recent electroencephalography (EEG) studies in both nulliparous women (Rutherford *et al.*, 2017) and mothers (Peltola *et al.*, 2018). Rutherford *et al.* (2017) found a stronger event-related potential (ERP; P300) in response to infant, but not adult faces after OXT administration. Peltola *et al.* (2018) observed stronger ERPs toward both infant and adult faces after OXT, whereas this effect was only observed for the N170, an earlier component for which Rutherford *et al.* (2017) observed no such effect, a difference that might be accounted for by the used of mothers and nulliparous women in the separate studies. Overall, these effects might relate to faster detection of infant faces after OXT, which has recently been observed (Holtfrerich *et al.*, 2016).

Although little can be inferred from these few studies because of different participants groups, methods, and setup, the findings are in line with the general notion derived from animal work that OXT facilitates social reward processing (Bos *et al.*, 2012; Bethlehem *et al.*, 2014; Rilling and Young, 2014; Patin *et al.*, 2018). Work in humans on social reward processing however shows strong person and context dependency (Bartz *et al.*, 2011; Wigton *et al.*, 2015; Wang *et al.*, 2017). For example, although OXT administration increased activation of the VTA during anticipation of social reward (i.e. happy faces) in healthy young women (Groppe *et al.*, 2013) and in police officers with posttraumatic stress disorder (PTSD; Nawijn *et al.*, 2016), it had opposite effects in the control group of the latter study (police officers without posttraumatic stress disorder (PTSD). Also, in a large study investigating the effect of OXT on social cooperation, OXT increased striatal (caudate and putamen) responses to cooperative interactions in men, whereas it decreased such responses in women (Feng *et al.*, 2015). A follow-up study by the same group replicated and extended this finding by also observing reduced VTA responses selectively in women after OXT administration (Chen *et al.*, 2017). These findings further demonstrate the need to take into account such individual differences in OXT administration studies (Bartz *et al.*, 2011; Bos *et al.*, 2012).

With respect to infant caretaking, an important factor that is likely to account for variation in neural responses to OXT is individual variation in caregiving motivation (Buckels *et al.*, 2015; Hofer *et al.*, 2017). Indeed, a recently validated questionnaire that taps into this concept, the Parental Care and Tenderness (PCAT) scale (Buckels *et al.*, 2015) and especially the nurturance subscale (PCAT-n) (Hofer *et al.*, 2017), predicted the motivation to look at infant faces in both parents and nulliparous adults. It has been proposed that care motivation positively relates to neural reward responses to infant faces (Glocker *et al.*, 2009b; Buckels *et al.*, 2015), but this has not yet been investigated. Therefore, in the current fMRI study we investigated the effect of OXT administration (24 IU) and parental care motivation on neural responses toward infant faces of varying cuteness in nulliparous women. Our paradigm was equivalent to Glocker *et al.* (2009b) in which nulliparous women were presented with pictures of infant faces that were adapted to have either enhanced or reduced baby schema. This resulted in increased activation of reward-related neural regions in response depending on cuteness condition. Since the current fMRI study is the first to use this paradigm while administering OXT and differences of OXT administration have been observed for participant’s sex and parental status (e.g. Feng *et al.*, 2015; Wigton *et al.*, 2015; Rutherford *et al.*, 2017; Wang *et al.*, 2017; Peltola *et al.*, 2018), only nulliparous women were included, to stay in line with Glocker *et al.* (2009b). Also, we only included women using oral contraceptives (OCs), as in these women the endogenous cyclic endocrine fluctuations which could interact with OXT administration are absent (Montoya and Bos, 2017).

First, we hypothesized that more pronounced baby schema increases neural activation of reward areas including the VTA and the striatum [caudate, putamen and nucleus accumbens (NAcc)] and that OXT increases this neural activation toward infant stimuli. Second, we hypothesized that these effects depend on individual variation in parental care motivation. Although we focus our analyses on neural regions involved in reward processing, we also included the amygdala, insula and ACC in our analyses. Several studies have observed effects of OXT in these regions in response to social stimuli such as faces (Bos *et al.*, 2012) as well as in response to infant vocalizations such as crying (Riem *et al.*, 2011), demonstrating the potential relevance of these regions for human caregiving behavior (Rilling, 2013; Feldman, 2015).

## Materials and methods

### Participants

Twenty-six right-handed healthy young nulliparous women were recruited at the university campus of Utrecht University to partake in the study. Only women using combined OCs (i.e. containing ethinylestradiol and levenorgestrel; Microgynon30) were included to avoid hormonal fluctuations during the cycle, and no scans were performed during menstruation (Montoya and Bos, 2017). Participants had no history of psychiatric, neurological, or endocrine abnormalities. Participants did not smoke and used no medication other than OCs. Participants were informed not to drink alcohol or use drugs 24 h and not eat or drink for 1 h prior to study participation. With respect to the drug administration, appointments were rescheduled when participants experienced a blocked or running nose. The experimental protocol was approved by the ethics committee of the University Medical Centre Utrecht and in accordance with the latest declaration of Helsinki. Participants gave written informed consent prior to participation and received payment afterwards. Because of technical problems during the fMRI session (artifacts due to scanner issues, *n* = 2; malfunctioning of the presentation software, *n* = 1) the final sample consists of 23 women (mean age, 20.2; s.d., 1.4, range, 19–24 years).

### Procedure

Participants were scanned at the same time of day on 2 separate days with an interval of at least 72 h in a timeframe between 12:00 and 7:00 p.m. Before drug administration (see supplementary online material) participants were screened for alcohol and drug use, were given brief explanations of the task and gave written informed consent.

The participants then self-administered the nasal spray under supervision of the experiment leader and were seated in a waiting room until asked to proceed to the scanner. Participants were screened using an MRI checklist and a metal detector, and were instructed to position themselves on the scanner bed as comfortable as possible and to try to relax. Head movement was minimized by foam pads which were placed between theradiofrequency (RF)-coil and participant’s head. Instructions and task images were displayed on an MRI-compatible monitor positioned at the head end of the scanner visible via an angled mirror attached to the coil. Participants also received a button box in their right hand to rate the cuteness of the infant face. Further instructions during the scan session were given by intercom. Average time interval between OXT administration and the start of the task was 49 min (s.d., 5.8), a time interval consistent with most studies showing effects of OXT on behavior published so far (Bos *et al.*, 2012). After the second session, participants were asked to guess which day they thought they received the OXT. They were not aware which day they received OXT or placebo (binomial: *P* > 0.05). Finally, participants were debriefed and given payment.

To control whether OXT had any effect on participants’ mood the Positive and Negative Affect Schedule (PANAS; Watson *et al.*, 1988) was filled out twice, once prior to administration upon arrival in the laboratory and once directly before proceeding to the scanner ($ \sim $30 min after administration). After the second day of testing, the participants received an e-mail to fill out on online versions of the PCAT questionnaire (Buckels *et al.*, 2015) that was used to calculate the nurturance scale (PCAT-n) based on Hofer *et al.* (2017). The PCAT measures parental care motivation (Buckels *et al.*, 2015) that consists of the conceptually separate constructs nurturance and protection, where the former uniquely predicts sensitivity for infant cuteness, whereas the latter predicts restrictive parenting practices and harsh moral judgments on moral transgressions (Hofer *et al.*, 2017). In the validation study Cronbach’s α of the PCAT-n was 0.88 (Hofer *et al.*, 2017) and for the current sample we obtained a Cronbach’s α of 0.75. Mean score on the PCAT-n was 3.69 (s.d., 0.74; range, 1.83–4.83).

### Infant face task and behavioral data analysis

To assess neural responses to infant stimuli we created a task based on Glocker *et al.* (2009b) in which the participants were presented with infant faces of varying levels of cuteness. The stimuli have been used in previous EEG research (Endendijk *et al.*, 2018) and consisted of nine different pictures of an infant faces (which comprised the normal condition), which were manipulated to create additional low-cuteness and high-cuteness condition of the same face yielding three conditions with a total of 27 stimuli (Borgi *et al.*, 2014). Detailed information on the stimuli can be found in the supplementary online material. A total of 27 stimuli were presented for 3 s in random order twice, resulting in a presentation of 54 stimuli throughout the run, 18 in each condition. Between the stimuli, there was a variable intertrial interval that averaged 5.5 s (min, 3.5; max, 7.5 s) during which a fixation cross was presented. Participants were instructed to carefully look at the stimuli and, after the offset of the face, use the button box in their right hand to rate whether the presented face was ‘not very cute’, ‘cute’, or ‘very cute’. When positioned in the scanner, and directly before the task started, participants received instructions and performed five practice trials. The same task was performed in both test sessions. Stimulus ratings on a three-point scale were included in the fMRI paradigm to guarantee attention of the participants to the stimuli. Additionally, the ratings were used to perform an exploratory behavioral analysis, in which responses on the three-point scale (not very cute: 1, cute: 2, very cute: 3) to the stimuli, as well as reaction times, were averaged per condition and checked for outliers and normality. Reaction times below 200 ms were removed from the data. The data were entered into separate 2 (administration) × 3 (cuteness) analyses of variance (ANOVAs). For the statistical analyses of the behavioral data, SPSS 23 (IBM analytics) was used and an α of 0.05 was applied to determine significance throughout the analyses.

### fMRI data analyses

Scanning parameters are reported in the supplementary online material. Preprocessing and subsequent analyses were performed with SPM12 (http://www.fil.ion.ucl.ac.uk/spm). Functional scans of both sessions were motion corrected after which the anatomical scan was then coregistered to the mean functional scan. Subsequently, using unified segmentation, the structural scan was segmented and normalization parameters were estimated. Using these normalization parameters, all volumes were normalized to a standard brain template (MNI) and were resliced at 2 mm isotropic voxel size. Smoothing with a 6 mm full width at half maximum Gaussian kernel was applied to the normalized functional volumes. Next, within both sessions, a general linear model (GLM) was applied to the data to investigate the effects of stimulus conditions. Neural responses to the different cuteness conditions were modeled using a 3 s boxcar function convolved with a hemodynamic response function (hrf) as implemented in the SPM12 software. Additional regressors of no interest which are entered into the analyses to reduce unexplained variance in the data include realignment parameters, a discrete cosine transform high-pass filter with a cutoff of 128 s and an hrf-convolved onset of the button press by which the participant rated the stimuli.

The contrast maps of the different cuteness conditions *vs* baseline in both sessions were entered in a second-level factorial ANOVA, with drug (OXT *vs* placebo) and stimulus (low, normal and high cuteness) as within-subjects factors. ComparativeT-tests were performed to investigate the (de)activations of all stimuli *vs* rest and the effect of OXT administration. F-tests were run for the effect of cuteness conditions and for the interactions of OXT administrations with cuteness. To control for multiple comparisons in the whole-brain analyses a threshold was set at *P* < 0.05 [family-wise error (FWE) corrected]. In addition, small volume corrections (SVC; *P* < 0.05 FWE) were applied for the predefined regions of the interest (ROIs): the amygdala, putamen, caudate, insula and ACC as based on the automated anatomical labeling (AAL) template (Tzourio-Mazoyer *et al.*, 2002). The VTA and NAcc are not included in the AAL template as separate masks and were therefore derived from previous empirical papers. The mask for the VTA was based on Groppe *et al.* (2013) and consists of 2 spheres of 10 mm radius around MNI coordinates ±9, −18 and −18. The bilateral mask for the NAcc was obtained from Montoya *et al.* (2014).

Furthermore, to investigate the effect of the PCAT-n on the neural responses toward infant faces and the interaction of the PCAT-n with OXT administration, we added participants’ scores on the PCAT-n as a covariate in a separate factorial whole brain analysis. Finally, for all the predefined anatomical ROIs that showed significant effects in the whole brain analyses, we extracted percent signal change using MarsBaR (Brett *et al.*, 2002) to further specify and visualize the effects. To test the robustness of the reported effects from the whole brain analyses, extracted values from the ROIs were entered into separate 2 × 3 ANOVAs, with administration (OXT and placebo) and condition (high, normal, and low cuteness) as within-subjects factors. As a control measure, order was added as a between-subjects factor, which was removed if it did not reach significance. Finally, to investigate brain behavior correlations, averaged behavioral ratings of the separate conditions were entered into exploratory correlational analyses together with the extracted values of the ROIs.

**Fig. 1:**
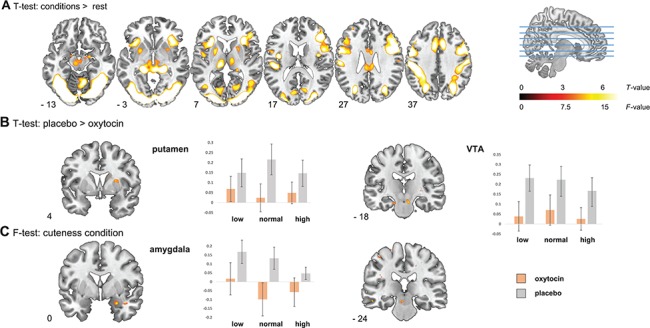
**A,** Axial slices with corresponding Y-coordinates (MNI) from the T-map of neural activation of all infant faces *vs* rest overlaid onto a standard anatomical template. **B,** Coronal slices with corresponding X-coordinates (MNI) from the T-map of neural deactivation toward all infant faces for oxytocin *vs* placebo, depicting significant deactivation in the VTA (right) and putamen (left). **C,** Coronal slices with corresponding X-coordinates (MNI) from the F-map of the effect of cuteness condition of the stimuli in both administration conditions. Significant modulation by cuteness was observed in the VTA (right) and amygdala (left). For the VTA, putamen and amygdala bar graphs of the parameter estimates extracted from anatomical bilateral ROIs in all conditions *vs* rest are displayed. Accompanying statistics are described in the text. All statistical maps are thresholded at *P* = 0.001 uncorrected, for illustration purposes only. Non-thresholded statistical maps of the main fMRI analyses can be found at the NeuroVault data repository (http://neurovault.org/collections/3699/).

**Table 1:** Peak *T*- and *F*-values, *P*-values, cluster sizes and MNI coordinates for significantly activated voxels

Experimental effect		Peak voxel location			*T*/*F*-value	Cluster size	*P*-values
Region		*x*	*y*	*z*		Voxels	
**Full factorial:**							
**T-test: stimuli > rest**							
Lingual gyrus	L	−24	−86	−16	23.09	9870	<0.001^*^
Fusiform gyrus	L	−34	−84	−14	22.97	s.c.	<0.001^*^
Lingual gyrus	R	20	−84	−10	22.48	s.c.	<0.001^*^
Supplementary motor area	L	−2	−2	54	14.22	9747	<0.001^*^
Precentral gyrus	L	−38	−20	56	14.00	s.c.	<0.001^*^
		−48	−24	52	13.55	s.c.	<0.001^*^
Hippocampus	R	18	−32	−2	12.98	250	<0.001^*^
		4	−32	−4	7.85	s.c	<0.001^*^
Hippocampus	L	−18	−32	−2	11.97	250	<0.001^*^
Thalamus	L	−12	−22	8	6.83	s.c.	<0.001^*^
Superior parietal lobule	L	28	−54	48	11.02	1949	<0.001^*^
		24	−60	54	10.25	s.c.	<0.001^*^
		30	−72	32	9.51	s.c.	<0.001^*^
Precentral gyrus	L	−56	2	34	9.91	375	<0.001^*^
		−40	0	32	5.52	s.c.	<0.001^*^
Rolandic operculum	L	−40	−6	14	9.75	86	<0.001^*^
Insula	L	−28	24	6	8.99	273	<0.001^*^
Calcarine sulcus	L	−14	−74	12	7.05	82	<0.001^*^
Rolandic operculum	R	40	−4	14	6.75	20	<0.001^*^
Fusiform gyrus	R	32	−14	−36	6.37	25	<0.001^*^
Calcarine sulcus	R	16	−70	12	6.20	49	<0.001^*^
Putamen	L	−24	6	2	5.82	18	<0.01^*^
Medial frontal cortex	L	−46	24	32	5.45	3	<0.05^*^
ACC	R	4	2	28	4.49	21	<0.01^**^
	L	−8	22	30	4.23	19	<0.05^**^
VTA	L	−8	−18	−10	4.24	110	<0.01^**^
	R	0	−16	−14	3.81	s.c.	<0.05^**^
Amygdala	R	22	−6	−12	3.80	2	<0.05^**^
				
**T-test: rest > stimuli**							
Cuneus	R	10	−86	26	10.82	713	<0.001^*^
Angular gyrus	L	−44	−74	30	9.36	721	<0.001^*^
Middle temporal gyrus	L	−56	−6	−16	8.47	661	<0.001^*^
	L	−26	26	44	7.92	344	<0.001^*^
Calcarine sulcus	L	−12	−58	12	7.84	1402	<0.001^*^
Medial orbitofrontal cortex	R	2	48	−6	7.78	1179	<0.001^*^
Lingual gyrus	R	10	−72	−4	7.51	76	<0.001^*^
Fusiform gyrus	R	34	−46	−4	7.39	86	<0.001^*^
ACC	L	−4	54	0	7.38	315	<0.001^*^
	R	4	26	−6	5.89	9	<0.001^*^
	R	8	50	8	5.15	2	<0.001^*^
Lingual gyrus	L	−10	−76	−6	7.35	41	<0.001^*^
Middle temporal gyrus	R	58	−6	−20	7.06	361	<0.001^*^
Parahippocampal gyrus	L	−32	−44	−6	6.81	115	<0.001^*^
Medial superior frontal cortex	L	−10	64	20	6.14	54	<0.001^*^
Inferior parietal cortex	L	−58	−40	42	6.12	61	<0.001^*^
Precentral gyrus	R	22	−30	64	5.68	17	<0.01^*^
Heschl gyrus	R	58	−2	6	5.66	10	<0.01^*^
Insula	L	−38	−20	−4	5.62	6	<0.01^*^
Superior temporal gyrus	L	−58	−6	6	5.48	11	<0.05^*^
Calcarine sulcus	R	30	−70	6	5.38	4	<0.05^*^
Postcentral gyrus	R	22	−40	58	5.29	3	<0.05^*^

*(continued).*

**Table 1(continued):** Peak *T*- and *F*-values, *P*-values, cluster sizes and MNI coordinates for significantly activated voxels

Experimental effect		Peak voxel location			*T*/*F*-value	Cluster size	*P*-values
Region		*x*	*y*	*z*		Voxels	
Superior frontal cortex	R	26	22	40	5.27	2	<0.05^*^
	R	60	−2	−6	5.24	1	<0.05^*^
	R	28	24	44	5.24	3	<0.05^*^
Caudate	L	−18	−6	28	5.17	1	<0.05^*^
**T-test: placebo > oxytocin**							
VTA	R	8	−18	−20	4.00	18	<0.05^**^
	L	−16	−16	−22	3.96	8	<0.05^**^
Putamen	R	26	4	12	3.91	19	0.05^**^
						
**F-test: cuteness condition**							
VTA	L	−4	−24	−16	9.66	5	<0.05^**^
Amygdala	R	26	0	−22	10.24	21	<0.05^**^
			−4	−18	10.03	s.c.	<0.05^**^

R, right; L, left; s.c., same cluster as above; ^*^whole brain FWE corrected at cluster level, ^**^small volume FWE corrected at cluster level.

**Table 2:** Peak *T*-values, *P*-values, cluster sizes and MNI coordinat

Experimental effect	Peak voxel location					*T*-value		Cluster size		*P*-values
Region		*x*	*y*	*z*				Voxels		
**Full factorial including covariate**										
**T-test: positive effect of PCAT-n**										
Inferior occipital cortex	L	−16	−94	−8		7.94		39		<0.001^*^
	R	28	−86	−14		7.45		55		<0.001^*^
	L	−36	−88	−6		7.25		14		<0.001^*^
	L	−22	−94	16		7.19		122		<0.001^*^
Middle occipital cortex	R	30	−88	6		5.95		13		<0.01^*^
Cuneus	R	14	−86	20		5.45		2		<0.05^*^
Superior temporal sulcus	R	58	−26	2		5.36		4		<0.05^*^
Superior temporal sulcus	R	42	−18	2		5.31		5		<0.05^*^
Precentral gyrus	L	−42	−8	56		5.15		1		<0.05^*^
Amygdala	L	−20	0	−12		3.45		5		0.053^**^
Insula	R	42	−16	4		5.24		46		<0.001^**^
	L	−42	−14	6		4.18		17		<0.05^**^
	R	−42	−2	4		4.06		57		0.058^**^
Putamen	R	22	14	6		4.21		40		<0.05^**^
	L	−26	6	4		3.88		19		0.057^**^
ACC	R	2	28	22		4.17		22		<0.05^**^
									
**T-test: interaction PCAT-n with drug admin**										
Putamen		−32	2	−4		4.11		12		<0.05^**^

R, right; L, left; s.c., same cluster as above; ^*^whole brain FWE corrected at cluster level, ^**^small volume FWE corrected at cluster level.

## Results

### Behavioral data

First, the data were checked for outliers based on the criterium of three times the s.d. above or below the mean in at least one of the conditions. Based on this criterium, one participant was excluded from the analyses on the rating data. Another participant was excluded from the analyses on the reaction times, leaving 22 participants in both analyses. As a next step, normality of the data was checked. For the ratings only the low cuteness condition in the OXT session was not normally distributed [*D*(22) = 0.231, *P* = 0.004]. For reaction times, the low-cuteness condition in both drug sessions significantly differed form normality [OXT: *D*(22) = 0.186, *P* = 0.047; placebo: *D*(22) = 0.208, *P =* 0.015]. Since the assumption of normality was violated for these variables, nonparametric statistical control analyses were performed in addition to the repeated measures ANOVAs.

Analyses on the rating data revealed no effect of administration [*F* (1, 21) = 0.34, *P* = 0.57, η_p_^2^ = 0.02] or interaction of administration with cuteness condition [*F* (2, 42) = 1.34, *P* = 0.27, η_p_^2^ = 0.06]. There was, however, a significant effect of condition [*F* (2, 42) = 70.82, *P* < 0.001, η_p_^2^ = 0.77]. All five pairwise comparisons between the three conditions were significant (mean rating low cuteness, 1.44; normal cuteness, 1.90; high cuteness, 2.01; all *P* < 0.005), which validated the cuteness categories created by manipulation of the stimuli. Nonparametric control analyses using a Friedman’s ANOVA confirmed the outcome of the ANOVA, by showing a main effect of condition [χ^2^(5) = 64.43, *P* < 0.001], and follow-up Wilcoxon tests showed significant differences between all conditions (all *z* > −2.55, all *P* < 0.02) but no effects between drug conditions (all *z* < −0.81, all *P* > 0.42). In an additional analysis, we added the PCAT-n as a covariate in the ANOVA. This revealed a significant three-way interaction of the PCAT-n with administration and cuteness condition [*F* (2, 40) = 5.16, *P* = 0.01, η_p_^2^ = 0.21]. This effect was driven by an interaction between condition and the PCAT-n in the OXT condition [*F* (2, 40) = 7.41, *P* = 0.002, η_p_^2^ = 0.27], which was absent in the placebo condition [*F* (2, 40) = 0.37, *P* = 0.70, η_p_^2^ = 0.02]. Correlational analyses demonstrated that this interaction was caused by a positive correlation of the PCAT-n with ratings in the low-cuteness conditions (*r*_s_ = 0.25), but negative correlations in the high cuteness condition (*r*_s_ = −0.37). However, these correlations did not reach significance (*P* > 0.09).

Analyses of the reaction times (overall mean, 660.1; s.d., 227.0 ms) showed no significant effect of drug, condition or interaction of drug with condition (all *F*-values < 0.32, all *P* > 0.73). Also, Friedman’s ANOVA showed no significant effect for the factor condition [χ^2^(5) = 1.04, *P* = 0.96].

To control for a possible effect of OXT administration on participants’ mood, we entered sum scores on the positive and negative scale of the PANAS (Watson *et al.*, 1988) in a 2 × 2 ANOVA with administration and time of measurement (before and after administration) as separate factors. The outcome shows that administration of OXT did not affect scores on these scales—positive scale [*F* (1, 22) = 0.04, *P* = 0.85, η_p_^2^ = 0.00] and negative scale [*F* (1, 22) = 0.17, *P* = 0.68, η_p_^2^ = 0.01]—or interacted with time of measurement—positive scale [*F* (1, 22) = 1.24, *P* = 0.28, η_p_^2^ = 0.05] and negative scale [*F* (1, 22) = 3.05, *P* = 0.10, η_p_^2^ = 0.12]. Overall mean scores on the PANAS were 12.75 (s.d., 2.88) for the negative scale and 33.98 (s.d., 5.77) for the positive scale.

### Imaging data

Whole brain analyses on all participants showed widespread activation across all infant stimuli *vs* rest, including bilateral visual cortices, hippocampus and sensory-motor areas. Of the ROIs, the bilateral VTA, amygdala, insula, ACC and putamen all showed significant activation levels, whereas no activation was observed for the caudate and NAcc (see Figure 1A, Table 1). Whole brain T-tests comparing the OXT and placebo condition over all cuteness conditions revealed that OXT administration resulted in reduced activation of the bilateral VTA (*P* < 0.05, SVC) and the right putamen (*P* = 0.05, SVC). In addition to this effect of administration, an effect of cuteness condition was observed in the left VTA (*P* = 0.05, SVC) and the right amygdala (*P* = 0.05, SVC; Figure 1C, Table 1). There was no significant increased activity after OXT *vs* placebo or interaction between OXT administration and cuteness condition in or outside any of the ROIs (Figure 1B, Table 1).

To further qualify and analyze the effects of cuteness in the amygdala and VTA, and drug administration in the VTA and putamen, we extracted the data from the anatomical ROIs and entered these in separate multivariate 2 × 3 ANOVAs with administration and condition as within-subjects factors. The results of these analyses confirmed our whole brain analyses (Figure 1), showing a main effect of OXT administration in the VTA [*F* (1, 22) = 9.17, *P* < 0.01, η_p_^2^ = 0.29] and putamen [*F* (1, 22) = 6.98, *P* = 0.02, η_p_^2^ = 0.24] and an effect of cuteness condition on the amygdala [*F* (2, 44) = 5.40, *P* < 0.01, η_p_^2^ = 0.20]. In addition, the analyses on the extracted values also revealed a significant main effect of administration on the amygdala [*F* (1, 22) = 4.59, *P* = 0.04, η_p_^2^ = 0.17], with higher activation in the placebo condition (mean = 0.116) compared to the OXT condition (mean = −0.046). Post-hoc pairwise comparisons showed that the main effect of condition in the amygdala was caused by a significant difference between responses to the low (mean, 0.093) and high (mean, −0.005) cuteness conditions (*P* = 0.01). The difference between low- and normal-cuteness condition (mean, 0.017) was not significant (*P* = 0.07), neither was the difference between normal and high cuteness condition (*P* = 1.0). In contrast, the effect of cuteness condition in the VTA, as observed in the whole brain analyses, was not significant in the extracted values. No other main effects or interactions were significant (all *P* > 0.1). Control analyses including order as a between-subjects factor showed no effect of order on the data (all *P* > 0.05) and left the effect of drug unaltered.

**Fig. 2:**
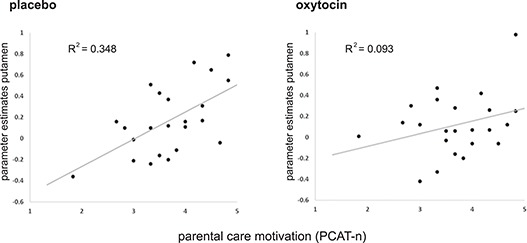
Scatterplots of the correlations between extracted parameter estimates from the anatomical bilateral putamen toward all infant stimuli and participants score on the PCAT-n. The left panel shows the significant correlation in the placebo condition (*r* = 0.60, *P* = 0.003), which was absent after OXT administration (*r* = 0.30, *P* = 0.16).

Next, we investigated the effect of individual differences in care motivation on the imaging data by adding the PCAT-n scores as a covariate in our whole brain analyses. Higher scores on the PCAT-n were significantly associated with increased neural activation toward all infant stimuli in several regions in the occipital cortex (all *P* < 0.001), the superior temporal sulcus (*P* < 0.05), the insula (*P* < 0.001, SVC), the ACC (*P* < 0.05, SVC) and the putamen (*P* < 0.05, SVC). In the amygdala this association did not reach significance (*P* = 0.053; Table 2). Critically, the PCAT-n also significantly interacted with OXT administration in the putamen (*P* = 0.03, SVC). This was not the case for the other ROIs. To qualify the interaction with OXT administration in the putamen, we computed correlations between individuals’ scores on the PCAT-n and extracted values of the bilateral putamen. This showed a strongly significant positive correlation between the PCAT-n and activation of the putamen toward all infant faces in the placebo condition (*r* = 0.60, *P* = 0.003; Figure 2), which was absent after OXT administration (*r* = 0.30, *P* = 0.16). This relation was significant in all three cuteness conditions after placebo, low (*r* = 0.57, *P* < 0.01), normal (*r* = 0.61, *P* < 0.01) and high (*r* = 0.50, *P* < 0.05), but not after OXT (all *r* < 0.42, all *P* > 0.05) Thus, whereas care motivation was positively associated with neural activation in the insula and putamen, this effect in the putamen was blunted after OXT administration.

### Brain behavior correlations

Since robust effects of cuteness were only observed in the amygdala, we ran exploratory correlational analyses to investigate whether subjective ratings of the different conditions in the scanner were related to neural activation in the amygdala. Only in the placebo condition, we observed a significant negative relation between subjective ratings and amygdala activation toward infant faces in the high cuteness condition (*r*_s_ = −0.43, *P* = 0.046). However, these correlational analyses were uncorrected for multiple comparisons and should thus be interpreted with caution.

## Discussion

The aim of this study was to investigate the effect of OXT on neural reward-related responses to infant faces of varying cuteness in healthy young nulliparous women. The results show that the infant stimuli elicited neural activation in brain areas previously related to reward and salience processing (Seeley *et al.*, 2007; Haber and Knutson, 2010; Rilling, 2013; Feldman, 2015), including the VTA, putamen, insula, ACC, and amygdala. Interestingly, no significant activation was observed in the caudate and NAcc, of which especially the NAcc is a core region in the reward pathway (Haber and Knutson, 2010). Furthermore, activation that was observed in the putamen, insula, ACC, and amygdala was positively related to a validated measure of caregiving motivation, the PCAT-n (Hofer *et al.*, 2017). This relation between participant’s nurturing motivation toward children and neural responses toward infant faces indicates that the current neuroimaging approach is useful in studying underpinnings of human caregiving behavior. Also, subjective ratings of the cuteness of the infant faces validated our manipulation of the baby schema, as all conditions differed in the expected direction (i.e. higher ratings for high-cuteness condition). Nonetheless, we could not replicate a previous finding that the high cuteness condition elicited stronger activation in the reward circuitry (Glocker *et al.*, 2009b), neither did we observe an effect of OXT on the subjective evaluations of the infant faces. We did observe an effect of our baby schema manipulation in the VTA and amygdala, but analyses on the extracted values indicated that the effect in the amygdala was driven by the low-cuteness infants eliciting more activation compared to the high cuteness condition, whereas the effect in the VTA was not significant in the extracted data from the anatomical ROI (Figure 1). That the stimuli did not elicit activation in the NAcc and caudate and that high cuteness stimuli did not elicit stronger activation in regions such as the VTA and putamen compared to low or normal cuteness condition might indicate that the neural responses observed do not reflect reward processing only but might relate to more general saliency of the stimuli. Because of conceptual overlap, the neural circuitry involved in saliency detection and reward processing are largely shared, and both contain the ROIs included in the current study such as the VTA, putamen, insula and amygdala (Seeley *et al.*, 2007; Haber and Knutson, 2010). This interpretation is in line with the increased activation for the low-cuteness condition in the amygdala, as that condition is rated as less cute and is also perceived more atypical (demonstrated by additional ratings from an independent group of participants reported in the additional online material), a feature that generally elicits amygdala activation (Todorov *et al.*, 2013). Thus, the infant stimuli elicited the hypothesized subjective responses and neural activation, which related to caregiving motivation, and may reflect overall salience of the stimuli, in addition to the rewarding value that the baby schema have (Kringelbach *et al.*, 2016). Future research could give insight into the specific contribution of cuteness perception, in addition to salience, on activation of the VTA by developing a neuroimaging paradigm in which cuteness and salience can be independently modeled. Comparable to research investigating the neural underpinning of trust evaluations (Todorov *et al.*, 2013), stimuli can be used that vary continuously on cuteness, instead of the categories used in the current study. A similar approach can also be employed to distinguish between cuteness and face typicality. Combining such faces with emotional information during a learning phase before the scan session could then be used to alter the relative salience of part of the stimuli (e.g. Bos *et al.*, 2016).

Critically, the study was set up to investigate the effect of OXT administration on neural responses to infant faces, and our results show reduced activation of the VTA, putamen and amygdala in response to infant faces. Furthermore, in the putamen the reduced activation after OXT administration also attenuated the relation between caregiving motives and putamen activation, which was observed in the placebo condition, but not after OXT administration (Figure 2). This finding could indicate that participants high in caregiving motivation are more susceptible to the effects of OXT, an observation that is in line with literature showing effects of experienced caregiving and representations of adult attachment on sensitivity to OXT administration (Bos, 2017). However, in is unclear why subjects with higher levels of caregiving motivation would show a stronger down-regulating effect in response to infants’ faces after OXT administration. Perhaps the phenomenon of regression to the mean is a more parsimonious explanation for this finding, in that the strongest responses to infant stimuli in the placebo condition are suppressed to the largest extent after OXT administration. Overall, the finding that OXT reduced neural activation in the VTA and putamen was against our predictions. These predictions were based on studies showing positive relations between OXT levels and activation of the ventral striatum toward own infant stimuli in mothers (Strathearn *et al.*, 2009) and increased activation of reward related areas after OXT administration in fathers toward own infant stimuli (Wittfoth-Schardt *et al.*, 2012; Li *et al.*, 2017) and in nulliparous young women toward infant (and erotic) pictures (Gregory *et al.*, 2015).

In retrospect, it might not be surprising that our findings are not in line with the studies in fathers, considering the sex differences that have been observed after OXT administration (Rilling *et al.*, 2014; Feng *et al.*, 2015; Wigton *et al.*, 2015; Wang *et al.*, 2017). Although OXT increased activation of the putamen in males during social cooperation, it decreased activation in the putamen and VTA in women (Chen *et al.*, 2017; Feng *et al.*, 2015). The study by Gregory *et al.* (2015) that found increased activation in the VTA in nulliparous women after OXT administration when observing infant stimuli differed considerably from our current setup. In that study, participants performed a one-back matching task and were presented with stimuli of crying and smiling infant, erotic stimuli and additional stimuli varying in valence, and OXT increased VTA responses only in response to erotic stimuli and crying infants (Gregory *et al.*, 2015). However, subjective ratings by the same participants showed that OXT also increased experienced arousal of the infant stimuli (Rupp *et al.*, 2013). That OXT selectively increased VTA responses toward crying but not smiling infants and that it increased arousal ratings of these stimuli indicate that the VTA responses in that study most likely do not reflect reward responses, but more generally the salience of the stimuli. This might also apply to the first study that demonstrated increased VTA responses after OXT in nulliparous women during anticipation of social reward (Groppe *et al.*, 2013), as in that study OXT had a similar effect during anticipation of punishment.

Irrespective of to what extent the VTA and putamen reflect the reward value of the presented infant faces, it is unclear why the effects of OXT vary between the studies. An important factor could be the emotional arousal inflicted by the stimuli, in which OXT up-regulates VTA and striatal responses toward social stimuli that elicit arousal (Groppe *et al.*, 2013; Gregory *et al.*, 2015), whereas less arousing reward stimuli, such as in the current stimuli or during social cooperation (Feng *et al.*, 2015; Chen *et al.*, 2017), down-regulate activation of these regions. That this effect also occurs in the context of human caregiving is interesting, as the observation that OXT reduces neural responses related to caregiving motivation nuances the view that OXT would have an overall positive effect on human parental behavior (Feldman, 2015).

In addition to differences in emotional arousal elicited by the stimuli of the current and previous studies, another factor might also have affected variation in neural activation after OXT administration, that is, the use of hormonal contraceptives. In the current study we only included women using combined OCs to control for cyclic endocrine fluctuations, whereas previous studies reporting increased VTA responses after OXT included only naturally cycling women (Groppe *et al.*, 2013; Gregory *et al.*, 2015). A recent study that compared naturally cycling women with those using OCs found that in response to pictures of their partner, OXT increased striatal and VTA activation, but only in the naturally cycling women (Scheele *et al.*, 2015). Use of OCs in the current sample might therefore have affected neural responsiveness to the administration, which can occur via down-regulating effects on estradiol and the OXT system (Bos *et al.*, 2012; Bos, 2017). In addition, by down-regulating estradiol, OCs can affect OXT action indirectly by lowering dopamine functioning (Almey *et al.*, 2015; Montoya and Bos, 2017), as dopamine–OXT interactions are considered critical for the role of OXT in the processing of social salience (Shamay-Tsoory and Abu-Akel, 2016). As it is currently unclear to what extent the effect of OXT depends on dopamine and estradiol (e.g. Striepens *et al.*, 2014), methods such as positron emission tomography and spectroscopy should be used to give more insight into the biological mechanisms of OXT administration. Furthermore, it is important that future studies employing an OXT administration paradigm include only naturally cycling women, or at least take the use of hormonal contraceptives into account in the analyses (Montoya and Bos, 2017). More broadly, the effects of OXT on neural responses toward infant faces of varying cuteness should also be investigated in mothers, fathers and nulliparous males, to investigate to what extent the current findings apply to other participant groups.

In addition to the currently observed effects in the VTA and putamen, analyses on the extracted values showed that OXT also attenuated neural responses toward the infant stimuli in the amygdala. Although we are the first to show this effect toward stimuli of infant faces, the effect seems in line with several previous findings of reduced amygdala activation after OXT in women (Bethlehem *et al.*, 2013), also in response to infant stimuli such as crying (Riem *et al.*, 2011). Nonetheless, a recent meta-analyses on studies involving effects of intranasal OXT on amygdala responses to emotional facial expressions found that most studies in women report increased activation of the amygdala after OXT (Tully *et al.*, 2018), and a similar effect was observed for women exposed to socially threatening scenes (Lischke *et al.*, 2012). It thus appears that effects of OXT administration on the amygdala, similar to those on the VTA and striatum, are strongly stimulus dependent. In this respect, future studies can benefit from including face categories other than infants only, to investigate to what extend the currently observed effects of OXT depend on infant faces, or could also be observed in adult faces that vary in cuteness (Peltola *et al.*, 2018), or even in other species (Holtfrerich *et al.*, 2016).

Overall, the current study is the first to report a down-regulating effect of OXT administration on neural responses toward infant faces related to caregiving motivation in nulliparous women. Thereby, it shows that also with respect to caregiving motivation, the effects of OXT may depend on specific personal characteristics (Bartz *et al.*, 2011; Bos, 2017), and as such our findings add to the literature that attempts to specify the role of OXT in how humans process social-emotion information (e.g. Bos *et al.*, 2012; Churchland and Winkielman, 2012; Guastella and MacLeod, 2012; Bethlehem *et al.*, 2014; Shamay-Tsoory and Abu-Akel, 2016; Patin *et al.*, 2018).

## Funding

The work in this paper was supported by a grant from the Netherlands Society of Scientific Research to P.A.B. (451-14-015).
